# Androgen excess uncouples circulating and hepatic lipid homeostasis in females on a high-calorie diet

**DOI:** 10.1016/j.jlr.2026.101031

**Published:** 2026-03-30

**Authors:** Serene Joseph, Vaibhave Ubba, Raniya Kalim, Selin Gunaydin, Mehak Dass, Danielle Waheed, Dalton Hilovsky, Joshua Hartsell, Jack Shpargel, Xiaojing Liu, Juncheng Wei, Ling Yang, Xiaofeng Yang, Hong Wang, Sheng Wu

**Affiliations:** 1 Center for Metabolic Disease Research, Temple University School of Medicine, Philadelphia, PA, USA; 2 Department of Molecular and Structural Biochemistry, NC State University, Raleigh, NC, USA; 3 Department of Medical Genetics and Molecular Biochemistry, Lewis Katz School of Medicine at Temple University, Philadelphia, PA, USA; 4 Department of Pediatrics, Johns Hopkins University School of Medicine, Baltimore, MD, USA

**Keywords:** androgen excess, androgen receptor, TG, GH, insulin resistance, hyperlipidemia

## Abstract

Androgen excess is a common feature of polycystic ovary syndrome, congenital adrenal hyperplasia, gender affirming hormone therapy and is a known driver of disrupted glucose homeostasis. However, its impact on hepatic lipid regulation remains poorly understood with conflicting results. In age and weight matched female mice, dihydrotestosterone (DHT) treatment under a regular chow diet did not alter hepatic or circulating lipid levels. In contrast, under a western diet, DHT protected against metabolic dysfunction–associated steatotic liver disease by reducing hepatic triglyceride (TG) accumulation, even as it exacerbated systemic hypertriglyceridemia. These effects occurred without changes in hepatic insulin–Akt signaling and were independent of hepatic androgen receptor signaling. Multiomics profiling revealed that DHT reprogrammed hepatic transcription toward a male like pattern driven by pulsatile growth hormone (GH)–STAT5 signaling. DHT enhanced hepatic STAT5 activation, suppressed expression of the fatty acid (FA) transporter Cd36, and reduced hepatic FA uptake, even in the presence of elevated circulating free fatty acid. DHT also increased VLDL-TG secretion and altered hepatic FA composition. Continuous GH infusion, which mimics the female GH secretion pattern, reversed these effects by attenuating STAT5 activation and restoring Cd36 expression. Together, these findings identify a GH-dependent, hepatic androgen receptor -independent pathway through which androgen excess shapes hepatic lipid homeostasis. They further show that DHT elicits divergent systemic effects, with impaired glucose homeostasis and enhanced lipolysis occurring alongside reduced hepatic lipid accumulation but increased TG secretion.

## Graphical Abstract

**Figure:**
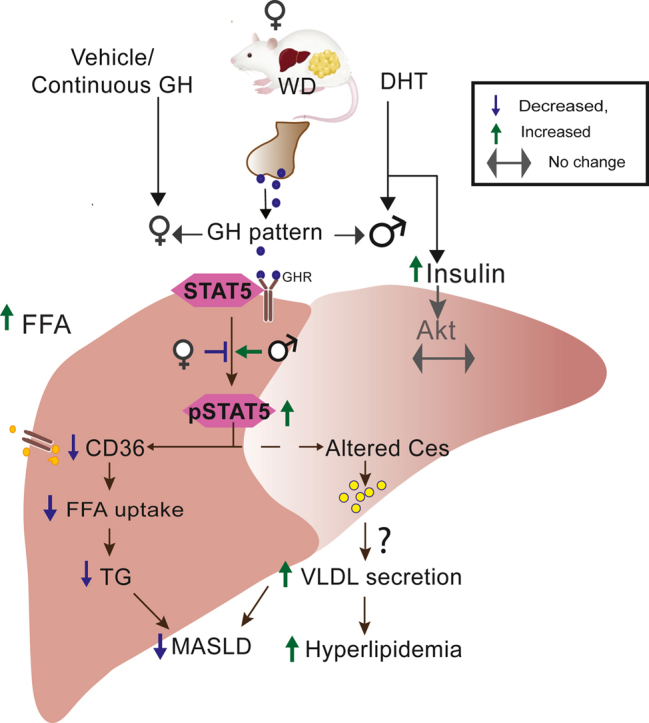

Androgen excess (AE) is a common endocrine problem observed in women with polycystic ovary syndrome (PCOS) (1, 2), congenital adrenal hyperplasia (3), and in individuals receiving gender affirming testosterone therapy (4, 5). Elevated androgen levels are strongly associated with impaired glucose homeostasis, insulin resistance, and dyslipidemia in the above conditions (1, 2, 3, 4, 5, 6). These clinical associations are also seen in animal models of AE which are further exacerbated by increased body weight (BW) and high fat diet exposure (7, 8, 9). Despite these consistent effects on systemic glucose and lipid regulation, the impact of AE on hepatic lipid metabolism remains unclear.

Clinical and experimental studies report conflicting hepatic outcomes in the setting of AE. In individuals receiving testosterone therapy or those with congenital adrenal hyperplasia, AE does not appear to cause clinically significant metabolic dysfunction associated steatotic liver disease (MASLD) (10, 11). Although MASLD prevalence is higher in some individuals with PCOS (12, 13, 14), this may reflect secondary factors such as obesity (15, 16) or developmental fat redistribution (17) as seen in animal models (18). Rodent models with hyperandrogenism also show inconsistent MASLD phenotypes (9, 19, 20), steatosis is typically observed at higher dihydrotestosterone (DHT) doses (>4 fold increase over control) and is often accompanied by increased body weight (BW), which itself promotes hepatic lipid accumulation (21, 22). Moreover, developmental testosterone excess (23) and intrauterine growth restriction (18) predispose offspring to PCOS associated MASLD. These discrepancies underscore substantial gaps in our understanding of adult onset AE and highlight the need to clarify how AE regulates hepatic lipid homeostasis.

The mechanisms by which AE influences hepatic lipid metabolism including free fatty acid (FFA) uptake, de novo lipogenesis, oxidation, triglyceride (TG) secretion, and storage, remain incompletely defined. Our previous studies demonstrated that AE impairs hepatic insulin signaling under regular chow conditions, an effect abolished by hepatocyte-specific androgen receptor (AR) KO (HepARKO) (24). It remains unclear whether pathophysiologically relevant AE in adulthood contributes to MASLD development or what mechanisms underlie such effects. To address this gap, we examined how AE, underweight-matched conditions, regulates hepatic lipid metabolism in both regular diet (RD) and western diet (WD) settings. We hypothesized that DHT would induce a fatty liver phenotype, and we conducted extensive experiments to rigorously test this hypothesis with various doses of DHT under different treatment duration and diet conditions. Contrary to our initial hypothesis, we found that under caloric excess, DHT treated mice exhibited reduced liver lipid levels without alteration under RD. Our studies suggested that this phenotype is through growth hormone (GH)–STAT5–dependent regulation of hepatic lipid metabolism.

## Materials and Methods

### Animals

Female mice with a floxed AR (Ar fl/fl) as previously described (24) were used in this study. The methodology for generating, genotyping, and validating this mouse line has been previously described (24). All procedures were performed with the approval of the Institutional Animal Care and Use Committee at Temple University. Female mice (7 weeks old) were implanted with silastic pellets (Dow Corning, 0.04 mm inner diameter and 0.085 mm outer diameter; Thermo Fisher Scientific, Hampton, NH) filled with DHT (4–10 mm) or empty vehicle (Veh). Pellets were replaced every 4 weeks to maintain DHT levels (24). Mice were randomly assigned to 4 groups: Control-Vehicle (Con-Veh), Control-DHT (Con-DHT), hepatic ARKO-Vehicle (HepARKO-Veh), and hepatic ARKO-DHT (HepARKO-DHT).

### Diet

Female mice were maintained on either normal chow (LabDiets 5,053, 4.11 kcal/g, Indiana) or a western/NASH diet (WD; Research Diets D09100310, 40% fat [mostly palm oil], 20% fructose, 2% cholesterol, 4.49 kcal/g, NJ). Female mice are resistant to steatosis with high fat diet alone, but the addition of fructose in WD accelerates liver lipid accumulation. Animals were housed under a 12-h light/dark cycle with ad libitum access to water.

### Dietary conditions and duration of DHT treatment

Mice were implanted with 10 mm DHT (DHT) or empty Veh pellets and maintained on either regular chow (RD) or WD. In the RD study, animals were observed for either 8.5 weeks (short term) or 6 months (long term). In the WD study, mice were maintained on WD for 8.5 weeks; BW was recorded weekly, and glucose, insulin, and pyruvate tolerance tests (GTT, ITT, PTT) were performed at designated time points. Detailed methodology in Supplemental methods (SM).

In a separate WD cohort, mice were pre fed WD for 6 weeks before subsequently receiving DHT or Veh pellets for an additional 2.5 weeks (total WD duration: 8.5 weeks). At the study endpoint, mice were fasted overnight and euthanized. Serum and tissues were collected. Organ weights were normalized to BW.

### Continuous GH perfusion study

In a separate cohort, mice were fed WD for one week before implantation with either a DHT pellet alone or a DHT pellet in combination with a growth hormone (GH, Cat#: Z02192-1, GenScript USA Inc.) filled osmotic pump (RWD Cat# 1006W; or Alzet Cat# 2006), as described previously (25, 26). The osmotic pumps delivered GH continuously into circulation at 48 ng/ml/hour/mouse for the 6-weeks duration of the study.

### Comprehensive Laboratory Animal Monitoring System

To assess whole-body energy metabolism under conditions of calorie excess and elevated androgens, mice underwent indirect calorimetry using a Comprehensive Laboratory Animal Monitoring System (Columbus Instruments, Columbus, OH). Detailed method is in SM.

### Hormone and biochemical assays

Serum hormones and biochemical markers, including insulin (7h fasting), leptin (overnight fasting), FGF21, DHT, ALT (overnight fasting), prolactin, and GH were measured using commercially available ELISA or bead-based assays according to the manufacturers' instructions. HOMA-IR was calculated from fasting glucose and insulin values levels as previously published (27). Detailed information is provided in SM and Supplemental Table S1.

### Histology

Liver tissues were fixed, sectioned, and processed for H&E, Sirius Red, Oil Red O, and F4/80 immunostaining using standard protocols. F4/80-positive cells were quantified using Fiji/ImageJ. Detailed staining procedures, antibodies, and imaging conditions are provided in SM.

### Triglyceride, cholesterol and free fatty acid determination

TG, cholesterol and free fatty acids (NEFA/FFA) were measured using the Triglyceride Determination Kit (Sigma-Aldrich, Cat# TR0100) and the Cholesterol Assay Kit (Abcam, Cat# ab65390) and Free fatty acids (NEFA/FFA) Fluorometric Assay kit (Elabscience, Cat# E-BC-F039) following the manufacturers' protocols. Detailed methods are given in SM. The same cohort was measured within one plate. Treatment and duration are different between studies, and the absolute value cannot be compared.

### VLDL TG secretion and fatty acid uptake

Hepatic TG production was assessed in mice injected with Poloxamer-407(Sigma-Aldrich, Cat# 16758; 1,000 mg/kg). Ex vivo fatty acid uptake was measured by fluorescence after incubation with 1 μM BODIPY FL C16 (Thermo Fisher Scientific, Cat# D3821). Detailed methods are given in SM.

### Hepatic fatty acid composition

Liver tissue (∼30 mg) was extracted using ice-cold 80% methanol containing a [^13^C_16_]-palmitic acid internal standard, and LC-ready supernatant collection. FFA was analyzed on a Vanquish UHPLC coupled to an Orbitrap Exploris 480 mass spectrometer using a HILIC amide column. Data were processed with Sieve software (Thermo Fisher Scientific, https://tools.thermofisher.com/content/sfs/brochures/BR-63141-SIEVE-Software-for-Differential-Expression-Analysis.pdf), and FFA species were identified by theoretical m/z and retention times, and quantified relative to the internal standard. Detailed methods are given in SM.

### RNA sequencing and serum proteomics

RNA sequencing and serum proteomics were done on liver and serum samples collected from WD fed mice. For the purposes of biological pathway analysis, all genes with adj *P* value ≤ 0.05 were used. Detailed methods are given in SM.

### Quantitative RT–PCR

Total RNA was extracted from mouse liver using TRIzol reagent (Thermo Fisher Scientific Cat# 15596026) and reverse transcribed with the iScript cDNA Synthesis Kit (Bio-Rad, Cat# 1708890). qRT-PCR was performed using iTaq Universal SYBR Green Supermix (Bio-Rad, Cat# 1725120) on a CFX96 Real-Time PCR System (Bio-Rad). Relative gene expression was calculated using the ΔΔCt method and normalized to Rpl19 (28). Primer sequences are listed in Supplemental Table S2.

### Western blots

Mouse liver tissues were lysed in RIPA buffer (Thermo Fisher Scientific, Cat # 89900) supplemented with protease and phosphatase inhibitors (Thermo Fisher Scientific, Cat # A32959). Nuclear proteins were separated using the NE- PER kit (Thermo Fisher Scientific, Cat # 78833) supplemented with protease and phosphatase inhibitors. Serum samples (2 μl) were diluted (1:5), denatured, and subjected to western blots. Primary antibodies are listed in Supplemental Table S3.

### Statistical analysis

Data are presented as mean ± SEM. Comparisons between 2 groups were performed using an unpaired Student's *t* test. Two-way ANOVA was used to assess 2 factors and their interaction, followed by Tukey's post hoc test for multiple comparisons. The specific statistical method used for each analysis is indicated in the corresponding legend. Statistical significance was set at *P* ≤ 0.05. All analyses were performed using GraphPad Prism 9.0 (San Diego, CA).

## Results

### DHT treatment does not alter serum or hepatic lipid levels in RD fed mice

Mice maintained on RD were implanted with 10 mm DHT pellets for 8.5 weeks and euthanized in a fasted state to assess hepatic lipid accumulation. DHT levels were further confirmed as 332.3 ± 33.01 pg/ml (Con-Veh: 117.4 ± 35.51 pg/ml, ∼three fold). DHT treatment did not affect BW or liver weight (corrected to BW) under RD conditions. Similarly, liver TG levels and cholesterol, serum TG and cholesterol, serum alanine aminotransferase (ALT) remain unchanged following DHT treatment under RD conditions (Table 1).

**Table 1:** Adult female mice fed with WD and receiving DHT for 8.5 weeks developed steatosis

Weeks	8.5 weeks			
Diet	Reg		Ta	
	Veh	DHT	Veh	DHT
Bw (g)	22.73 ± 0.31^a^	22.92 ± 0.55^a^	23.55 ± 0.46^a^	24.58 ± 0.50^a^
Corrected liver weight (g/g)	0.043 ± 0.003^a^	0.041 ± 0.001^a^	0.051 ± 0.001^b^	0.046 ± 0.001^a^
liver TG (mg/g)	27.45 ± 3.08^a^	23.13 ± 2.71^a^	39.48 ± 2.76^b^	22.00 ± 2.77^a^
Liver Cholesterol (mg/g)	3.64 ± 0.10^a^	3.74 ± 0.30^a^	5.51 ± 0.31^b^	3.62 ± 0.13^a^
Serum TG (mg/dl)	57.61 ± 15.61^a^	60.08 ± 7.27^a^	57.61 ± 5.12^a^	102.5 ± 9.32^b^
Serum Cholesterol (mg/dl)	115.9 ± 15.09^a^	111.8 ± 8.55^a^	181.1 ± 9.35^b^	189.8 ± 11.41^b^
ALT (U/L)	38.62 ± 7.27^ab^	27.26 ± 4.97^b^	56.64 ± 4.38^a^	47.13 ± 7.31^ab^

Mice were fed either a RD (n = 4–8) or a WD (n = 8–18) and treated with vehicle (Veh) or DHT. Statistical analysis was performed using two-way ANOVA followed by Tukey's post hoc test. Data are presented as mean ± SEM. Groups sharing the same letter are not significantly different, whereas groups with different letters are significantly different (*P* ≤ 0.05).

WD, western diet.

To examine the long-term effects of DHT, a separate cohort of mice was treated with DHT for 6 months while maintained on RD (Supplemental Fig. S1A). No significant differences were observed in BW or liver weight (corrected to BW) (Supplemental Table S4). Histological analysis of liver sections stained with H&E and Oil Red O revealed no evidence of increased fat accumulation in DHT treated mice (Supplemental Fig. S1B, C). Quantitative biochemical assays showed no significant differences in hepatic TG and cholesterol content. Serum TG, cholesterol levels and ALT levels remained comparable in both Veh and DHT treated groups (Supplemental Table S4).

Overall, these findings demonstrate that both short-term and long-term DHT treatment under RD, does not alter BW or induce hepatic steatosis in female mice. Similar results were observed with the use of 4 or 6 mm DHT pellets (1.5–2 FC of DHT levels compared to Con-Veh, data not shown).

### DHT impairs glucose tolerance in WD fed female mice without changing body weight

**Fig. 1:**
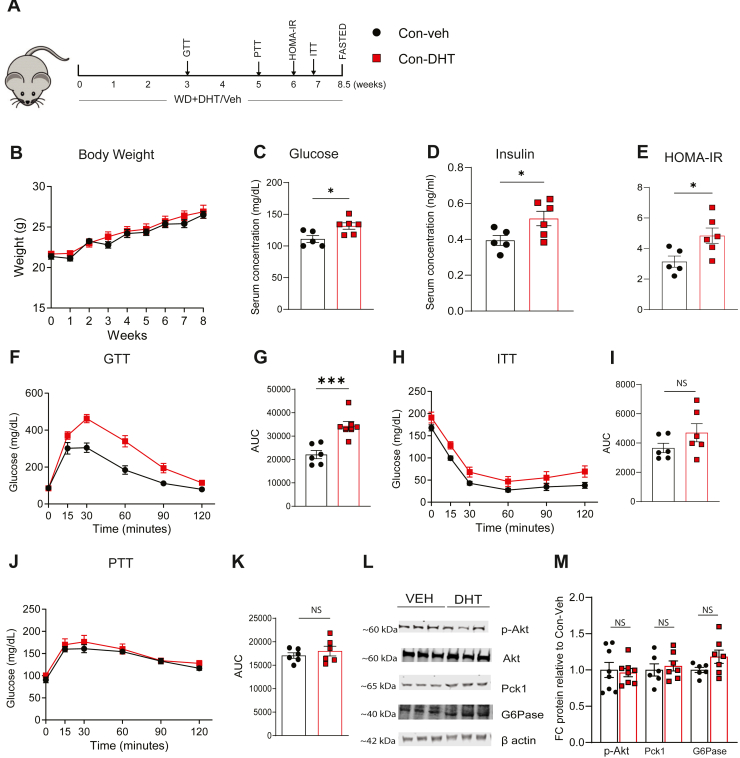
DHT impaired glucose homeostasis in WD fed mice (A) Schematic diagram: Ar floxed female mice on WD were treated with DHT or Veh for 8.5 weeks (B) Body weight over 8 weeks (C–E) Fasting (7 h) blood glucose levels, insulin levels, HOMA-IR. (F, H, J) Glucose tolerance test, Insulin tolerance test, Pyruvate tolerance test. (G, I, K) Area under curve (AUC) for glucose tolerance test, insulin tolerance test and pyruvate tolerance test respectively. (L–M) Representative western images, densitometric analysis of p-AKT, PEPCK, G6Pase respectively. N = 5–8. Statistical analysis was performed by Student's *t* test. Values are mean ± SEM. *P* ≤ 0.05; ∗*P* ≤ 0.05, ∗∗∗*P* < 0.001, NS, non significant; FC, Fold change; WD, western diet; DHT, dihydrotestosterone.

Female mice were fed a WD (contains high fructose and accelerates steatosis development compared to a high fat diet alone) and were treated with 10 mm DHT (Con-DHT) or vehicle (Con-Veh) for 8.5 weeks (Fig. 1A). Whole-body energetics (Table 2), including RER, heat production; activity, and food intake were unchanged between groups. BW (Fig. 1B) remained unaltered between Con-Veh and Con-DHT mice under WD over 8.5 weeks (correspondingly, there was no increase in corrected weight of gonadal fat and inguinal fat, but an increase in brown adipose tissue, *P* < 0.01, Supplemental Table S5). DHT mice exhibited disrupted glucose homeostasis as under RD (24). Fasting glucose levels were significantly elevated after 7 h of fasting in the DHT group (Fig. 1C). Correspondingly, fasting serum insulin levels were significantly increased; resulting in a marked rise in HOMA-IR values (Fig. 1D, E). Glucose tolerance was impaired in DHT treated mice, as indicated by significantly higher serum glucose levels and an increased area under the curve (AUC) (Fig. 1F, G). While the relative glucose levels were lower in the Veh group compared to the DHT in ITT, there was no difference in the incremental AUC when calculated from the respective baseline for each group (Fig. 1H, I). However, the PTT showed no significant differences between the groups (Fig. 1J, K). p-Akt, Pck1 (PEPCK encoded) and G6Pase, key proteins mediating hepatic insulin signaling and gluconeogenesis respectively, were unchanged (Fig. 1L, M). There was a trend toward increased GTT AUC in HepARKO-DHT compared with HepARKO-Veh mice (Supplemental Fig. S5A, B).

**Table 2:** Adult female mice fed with WD and receiving DHT for 8.5 weeks did not show changes in whole body energetics

	Con-Veh	Con-DHT	*P* Value
	Mean ± SEM	Mean ± SEM	
RER	0.89 ± 0.01	0.92 ± 0.01	NS
Heat (kcal/hr)	0.44 ± 0.01	0.47 ± 0.01	NS
X ambulatory	392.7 ± 78.24	332.7 ± 54.51	NS
Food intake (g)	6.06 ± 0.40	7.01 ± 0.70	NS

Whole body energetics were performed on mice fed a WD with DHT or Veh. Statistical analysis was done by Student's *t* test. (N = 7 each). Values are mean ± SEM (*P* ≤ 0.05).

NS, Non Significant; WD, western diet.

### DHT treatment induces hyperlipidemia but improves MASLD in WD fed adult female mice independent of hepatic AR

**Fig. 2:**
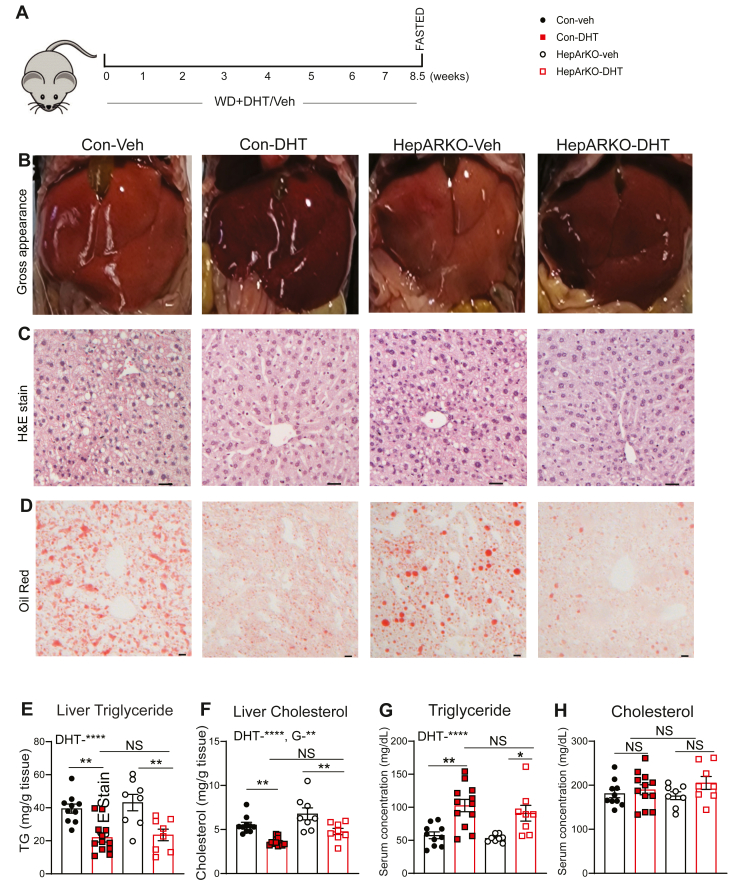
DHT treatment protected against metabolic dysfunction associated steatotic liver disease, but elevated serum triglycerides in WD fed mice independent of the hepatic androgen receptor (A) Schematic diagram: Ar floxed female mice on WD were treated with DHT or Veh for 8.5 weeks (B) Representative images of gross liver appearance and (C–D) H&E stain (20x), Oil Red (4x) staining in Con-Veh, Con-DHT, HepARKO-Veh, HepARKO-DHT mice. Scale bar: 20 μM. (E–F) Liver TG, cholesterol and (G–H) Serum TG, cholesterol in Con-Veh, Con-DHT, HepArKO-Veh, HepArKO-DHT mice. (N = 8–12). Statistical analysis was performed by 2-way ANOVA followed by Tukey's test. Data are presented as mean ± SEM. *P* ≤ 0.05; ∗*P* ≤ 0.05, ∗∗*P* < 0.01, ∗∗∗∗*P* < 0.0001, NS, non significant; WD, western diet; DHT, dihydrotestosterone.

Experimental set up is described in Fig. 2A. Mice were sacrificed after being fed a WD for 8.5 weeks. Liver (Table 1) weights were recorded. Female mice exhibited increased MASLD parameters on WD compared to RD (Table 1). Compared to Con-Veh female mice, the livers from 10 mm DHT treated (Con-DHT) appeared visually less pale (Fig. 2B, left 2 columns), and showed reduced fat accumulation by H&E and Oil Red O staining (Fig. 2C, D), with reduced liver weight (corrected to BW) (Table 1).

To determine the role of hepatic Ar in MASLD, the study was extended to include hepatic androgen receptor knock out (HepARKO) mice treated with Veh and DHT. In these mice, the livers of DHT treated animals (HepARKO-DHT) again appeared less pale, had reduced lipid droplet by H&E and Oil Red O staining compared to their HepARKO-Veh counterparts (Fig. 2B–D, right 2 columns).

The liver (Fig. 2E, F) and serum (Fig. 2G, H) TG and cholesterol levels were compared among Con-Veh, Con-DHT, HepARKO-Veh, and HepARKO-DHT groups. Con-DHT had reduced liver TG and cholesterol, but increased serum TG levels compared to Con-Veh (Table 1). Similar patterns were seen in HepARKO-DHT when compared to HepARKO-Veh (Fig. 2). There was no significant difference in liver and serum TG, liver cholesterol between Con-DHT and HepARKO-DHT groups, suggesting these changes were not mediated by hepatic AR (Fig. 2E–G). Additionally, liver fibrosis and inflammation as assessed by picrosirius red staining and F4-80 staining respectively (Supplemental Fig. 2A, B), were not altered by DHT under current conditions.

### Liver transcriptomics revealed a male-biased, GH–STAT5 driven hepatic gene expression profile

**Fig. 3:**
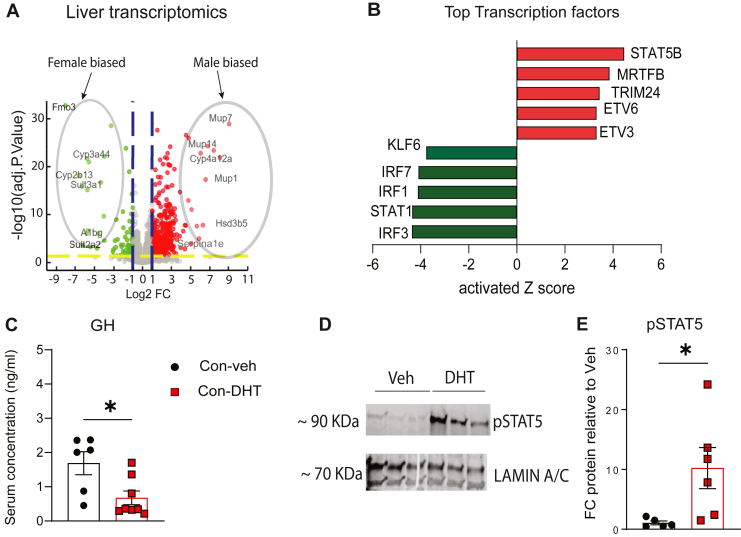
DHT treatment in WD fed mice shifts the hepatic transcriptome toward a male biased GH-STAT5 regulated gene expression pattern (A) Volcano plot of hepatic transcriptome changes induced by DHT. Circled areas indicate genes that are highly sexually dimorphic. N = 4. (B) Top upstream transcription factors from differntially enriched genes (DEG) in the livers treated with DHT. (C) Basal GH was reduced in DHT mice fed WD. (D, E) Representative Western blot and densitometric analysis showing activation of STAT5 in liver tissue treated ex vivo with 400 ng/μl GH for 15 min. N = 5–8. Statistical analysis was performed using Student's *t* test. Values are mean ± SEM. *P* ≤ 0.05; ∗*P* ≤ 0.05. FC, Fold change; WD, western diet; DHT, dihydrotestosterone; GH, growth hormone.

To determine how AE reprograms the female liver, RNA sequencing was performed on liver samples from Con-DHT and Con-Veh mice maintained on a WD. At a broader level, differentially enriched genes as depicted in the volcano plot (Fig. 3A) highlight a pronounced masculinization of hepatic transcriptome in DHT treated mice. Female-biased genes, including *Fmo3, Cyp2b13, Sult3a1, Sult2a2, A1bg*, and *Cyp3a44*, were significantly suppressed, whereas male-biased genes such as *Mup, Cyp4a12a, and Hsd3b, Serpina1e* were markedly induced (Fig. 3A). The top 20 upregulated and downregulated genes, many of which are strongly associated with sexual dimorphism, are presented in Supplemental Table S6 (29, 30). Differentially enriched gene were compared to sex biased genes (male and female livers) (25), and we saw an inherent overlap (∼20%), suggesting a shift in sex specific gene pattern in the liver.

All genes with an adj *P* value < 0.05 (N = 1,568), were used in downstream pathway analysis. With IPA analysis, the transcription factor STAT5b (major liver isoform of STAT5) was identified as the most activated upstream regulator, with an activation z-score of 4.47 (Fig. 3B). STAT5b mediates the altered transcription of sexually dimorphic genes and metabolism, and serum proteomics analysis (Supplemental Fig.3A, B) further supported sexual dimorphism. The most differentially regulated circulating proteins mirrored hepatic changes, including robust upregulation of MUPs and Serpina1e, with ∼50% of these proteins overlapping with those differentially expressed between male and female serum (31). Collectively, these findings suggest that DHT acts on the female liver through a STAT5b-regulated gene network that drives sexual dimorphism.

Given that STAT5b activity is tightly regulated by GH pulsatility, we next assessed GH signaling. GH basal levels were significantly decreased in DHT mice (Fig. 3C) as in male mice (32). Livers from Con-DHT mice showed higher pSTAT5 expression compared to Con-Veh mice when stimulated with GH acutely (ex vivo, Fig. 3–E). This is consistent with prior studies demonstrating that sexually dimorphic hepatic gene expression is regulated by STAT5 and shaped by growth hormone (GH) pulsatility under the influence of sex hormones (32, 33, 34, 35, 36).

### DHT treatment in WD fed mice decreased hepatic fatty acid uptake while increasing VLDL-TG secretion

To understand the functional consequences of DHT induced transcriptional reprogramming and identify pathways that may mediate its metabolic effects, all genes with adj *P* value < 0.05 were subjected to Gene Ontology and KEGG pathway analysis (SupplementaL Fig. 4A–D). Cellular component analysis showed that the ER exit site was most enriched category. Carboxylesterases family of genes were altered on our RNA -seq (Supplemental Table S7) and known to be involved in ER processing. Further, molecular and biological function analysis revealed significant enrichment in lipid transporter activity, lipid catabolic processes, fatty acid metabolic processes. Pathways related to retinol metabolism; biosynthesis of unsaturated fatty acids were overrepresented (including both upregulated and downregulated genes).

**Fig. 4:**
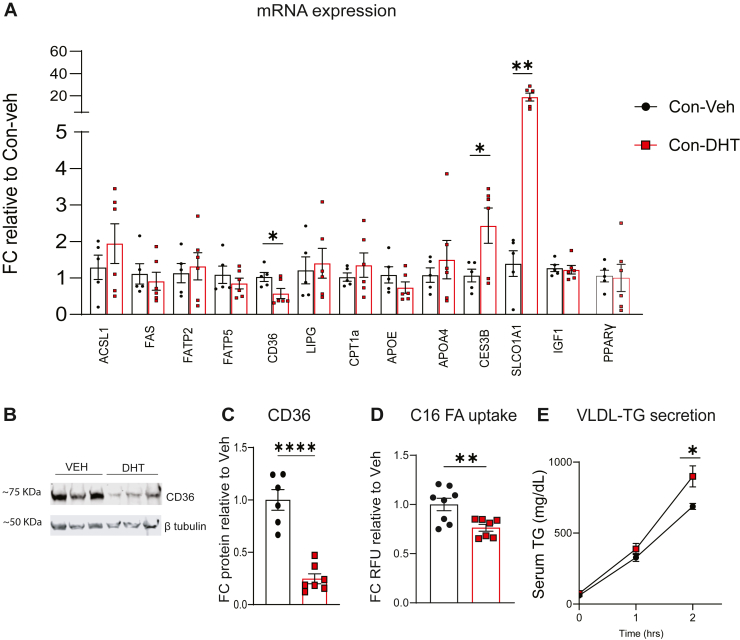
DHT treatment in WD fed mice decreased fatty acid uptake and increased VLDL-TG secretion (A) Gene expression was measured by qRT-PCR in livers of mice fed WD and treated with DHT. (B–C) Representative Western blot images, densitometric analysis of CD36 in livers of Con-Veh and Con-DHT mice. (D) C16 fatty acid uptake measured in liver lysates following ex vivo treatment. (E) VLDL-TG secretion following p-407 treatment. N = 5–9. Statistical analysis was performed by Student's *t* test. Values are mean ± SEM. *P* ≤ 0.05; ∗*P* < 0.05, ∗∗*P* < 0.01, ∗∗∗∗*P* < 0.0001. FC, Fold change; WD, western diet.

Given these pathway-level changes, we next investigated how DHT influences hepatic lipid regulation by measuring key genes with qRT-PCR (Fig. 4A). The genes studied included *Pparγ*, a central transcription factor in lipid metabolism; genes involved in fatty-acid uptake (*Cd36*, *Fatp2*, *Fatp5*); de novo lipogenesis (*Fas*); fatty-acid oxidation (*Cpt1a*, *Acsl1*); phospholipid hydrolysis (*Lipg*); and lipid secretion (*Apoe*, *Apoa4*, *Ces3b*). Genes were selected based on significant RNA-seq changes (adj. p < 0.05), with the exception of *Fatp2*, *Fatp5*, and *Pparγ*, which were included because of their established roles in hepatic lipid metabolism. *Igf1* was also assessed due to its involvement in GH signaling. Among these targets, we validated changes in *Cd36*, *Ces3b*, and *Slco1a1*, all of which are sexually dimorphic genes (37, 38, 39). DHT treatment significantly reduced *Cd36* mRNA expression, whereas *Ces3b*, which is involved in VLDL–TG secretion, and *Slco1a1*, a transporter implicated in bile acid metabolism, were increased. In contrast, *Pparγ*, *Fas*, *Cpt1a*, *Acsl1*, *Lipg*, *Apoe*, *Apoa4* and *Igf1* were unchanged by DHT.

At the protein level, Western blot analysis confirmed a significant reduction in hepatic CD36 in Con-DHT mice compared with Con-Veh (Fig. 4B, C), and a similar decrease was observed in HepARKO-DHT mice relative to HepARKO-Veh (Supplemental Fig. 5C, D). Ex vivo Bodipy-palmitate uptake was significantly reduced in Con-DHT livers compared to Con-Veh (Fig. 4D).

To evaluate lipid export, we measured VLDL-TG secretion following poloxamer-407 administration, an inhibitor of lipoprotein lipase. There was a significant increase in the secretion of the VLDL/TG at 2 h in the DHT mice, (Fig. 4E) following treatment with poloxamer-407. ANGPTL3, a lipase inhibitor and APOE, a TG remnant clearing apolipoprotein were not changed in the serum and liver tissue of mice receiving DHT (Supplemental Fig. S6).

Because BAT mass increased in DHT treated mice, we also examined *Cd36*, *Ces3b*, and *Slco1a1* expression in BAT; however, *Cd36* levels were unchanged (Con Veh FC: 1.56 ± 0.62 vs. Con DHT FC: 1.74 ± 0.45), and *Ces3b* and *Slco1a1* were expressed at negligible levels.

### DHT treatment altered hepatic fatty acid composition and increased serum FFA in WD fed mice

**Fig. 5:**
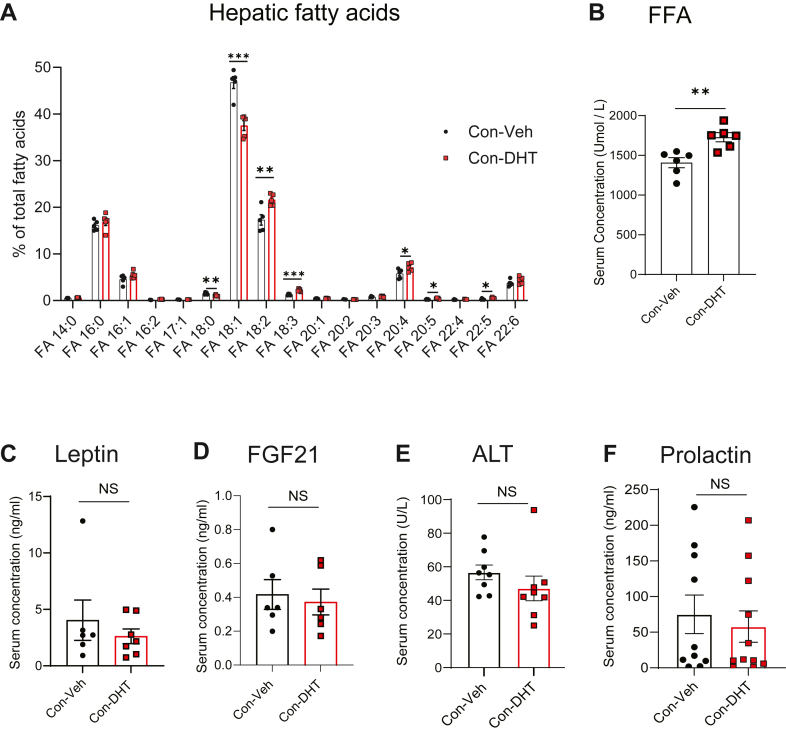
DHT treatment in WD fed mice altered hepatic fatty acid composition and decreased FFA in circulation (A) Hepatic fatty acid composition was measured by LC-MS. Serum (B) FFA (free fatty acid, fasted) (C) Leptin (fasted), (D) FGF21 (fed), (E) ALT in Con-Veh and Con-DHT mice. (F) Prolactin was measured by luminex in Con-Veh and Con-DHT mice. N = 6-11Statistics was performed using Studen'st *t* test. Values are mean ± SEM. *P* ≤ 0.05; ∗*P* ≤ 0.05, ∗∗*P* < 0.01, ∗∗∗*P* < 0.001, NS- non significant; WD, western diet; DHT, dihydrotestosterone; FFA, free fatty acid.

DHT treatment significantly reduced hepatic TG content, and because of this shift in overall lipid storage, we next examined liver fatty-acid composition. DHT decreased stearic acid (18:0) and oleic acid (18:1) while increasing the relative proportion of PUFAs-including linoleic acid (18:2), α-linolenic acid (18:3), arachidonic acid (20:4), eicosapentaenoic acid (20:5), and docosapentaenoic acid (22:5) (Fig. 5A). Furthermore, serum FFA levels were significantly increased in DHT treated mice compared with controls (Fig. 5B).

### Serum leptin and FGF21 were not changed in DHT mice fed WD

To assess the systemic effects of DHT treatment in the context of WD, we analyzed serum levels of key hormones involved in lipid metabolism. Leptin levels (Fig. 5C) and Fgf21 levels (Fig. 5D) remained unchanged in DHT treated mice compared to Veh treated mice. Similarly, serum ALT levels (Fig. 5E), a marker of liver function and hepatocellular injury, showed no significant differences. Prolactin levels (Fig. 5F) remained unchanged, indicating that DHT treatment did not significantly impact this hormone secretion from the pituitary.

### Continuous GH treatment reversed DHT protection against MASLD without altering HOMA-IR

**Fig. 6:**
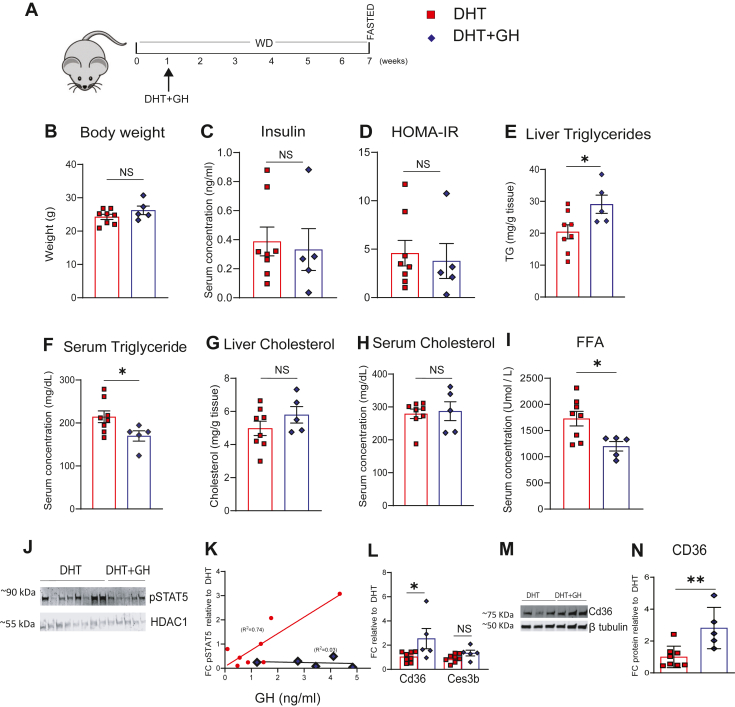
Continuous GH infusion increased liver TG in DHT mice fed with WD (A) Schematic diagram: Ar floxed female mice were treated with DHT or DHT + GH for 6 weeks after 1 week of WD initiation. (B) BW at sacrifice. (C, D) Serum insulin levels, HOMA-IR. (E, F) Liver, serum TG. (G, H) Liver, serum cholesterol. (I) Serum FFA (free fatty acid). (J, K) Immunoblot of pSTAT5 in nuclear extracts from DHT and DHT + GH livers, linear regression analysis of pSTAT5 in these samples. (L) Cd36, Ces3b mRNA (M, N) Representative western images, densitometric analysis of Cd36 n livers of DHT and DHT + GH mice. (N = 5–8). Statistical analysis was performed by Student's *t* test. Values are mean ± SEM. *P* ≤ 0.05; ∗*P* ≤ 0.05, ∗∗*P* ≤ 0.01, NS, Non significant; FC, Fold Change; WD, western diet; DHT, dihydrotestosterone; GH, growth hormone; FFA, free fatty acid.

To determine whether continuous, female-like GH secretion can reverse DHT's GH-dependent protection against MASLD, we examined hepatic lipid metabolism in DHT-treated mice. Experimental set up has been described in Fig. 6 A. Following 1 week of WD initiation, mice were treated with DHT or DHT + GH for a period of 6 weeks (as limited by the pump's maximum release duration). The groups showed no difference in BW (Fig. 6B), liver weight (corrected to BW) (DHT: 0.04 ± 0.00 vs. DHT + GH: 0.04 ± 0.00), levels of serum insulin and HOMA-IR between DHT and DHT + GH pump (Fig. 6C, D). Their average GH concentrations were DHT: 1.36 ± 0.47 ng/ml versus DHT + GH: 3.27 ± 0.62 ng/ml. Continuous GH infusion reversed DHT effects by increasing liver TG levels and decreasing serum TG levels in DHT + GH group (Fig. 6E, F) While the liver cholesterol levels were not significant, there was a trend towards an increase in liver cholesterol levels, but no change in serum cholesterol levels (Fig. 6G, H). There was a significant decrease in serum FFA levels in the DHT + GH group compared to the DHT group (Fig. 6I).

Since continuous GH infusion reversed liver and serum TG, we examined whether continuous GH delivery disrupts the pSTAT5–Cd36 signaling axis to produce these changes. pSTAT5 showed a linear increase in its expression with increasing concentration of GH (pulsatile pattern) in the DHT group (R^2=^ 0.74), while there was no linear relationship in the DHT + GH (GH continuous released pattern) group (R^2=^0.02) (Fig. 6J, K). There was a significant increase in the expression of Cd36 mRNA and protein levels in the livers of DHT + GH group compared to DHT group (Fig. 6L–N). However, *Ces3b* mRNA levels were not altered (Fig. 6L).

### Subsequent DHT treatment reverses WD induced MASLD in mice

**Fig. 7:**
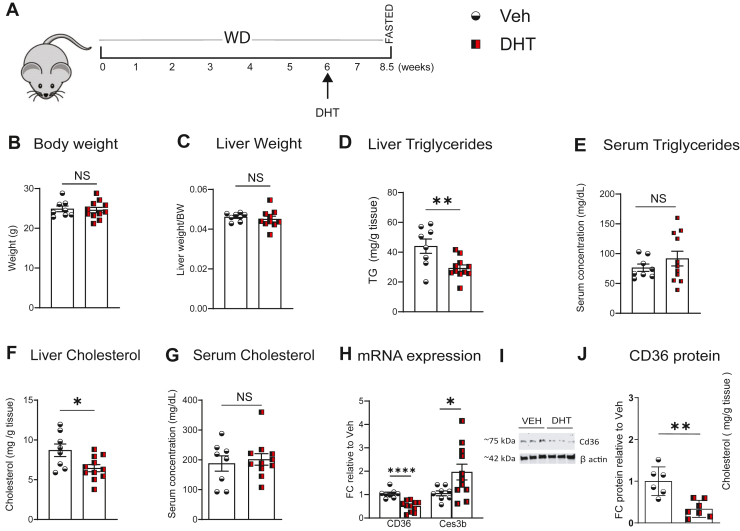
Subsequent DHT treatment decreased WD induced hepatic lipid accumulation and CD36 expression (A) Schematic Design: Ar floxed female mice were fed with WD for 8.5 weeks and treated with DHT or Veh during the last 2.5 weeks (B–C) BW and corrected liver weight. (D–E) Liver, serum TG and (E–F) Liver, serum cholesterol in Veh and DHT mice. (H) qRT-PCR of Cd36 and Ces3b. (I, J) Representative Western blot images, densitometric analysis of CD36 in livers of mice fed a WD and treated with DHT for last 2.5 weeks. N = 8–11. Statistical analysis was performed by Student's *t* test. Values are mean ± SEM. *P* ≤ 0.05; ∗*P* ≤ 0.05, ∗∗*P* < 0.01, ∗∗∗∗*P* < 0.0001, NS-non significant. FC, Fold Change; WD, western diet; DHT, dihydrotestosterone.

To determine whether androgen signaling can reverse established steatosis, we next examined the effects of DHT treatment in mice prefed with WD. Mice fed WD for 6 weeks were implanted with either DHT or Veh pellets for 2.5 weeks (Fig. 7A). BW and liver to BW ratio were not different between groups (Fig. 7B, C). Consistent with previous experiments, hepatic TG content was significantly reduced in DHT treated mice compared to Veh (Fig. 7D). However, serum TG levels were not significantly altered (Fig. 7E). Liver cholesterol levels were also significantly decreased (Fig. 7F), and serum cholesterol remained unchanged (Fig. 7 G).

qRT-PCR and Western blot analyses showed a significant reduction in hepatic Cd36 expression at both the mRNA and protein levels in the DHT group, indicating that DHT downregulates Cd36 in the liver (Fig. 7H–J), even after steatosis has been induced. Further, Ces3b mRNA is also increased in the DHT group (Fig. 7H).

Treatment and duration are different among Fig. 2, Fig. 5 and 6 and the absolute value among figures cannot be compared for the same assays.

## Discussion

Women with AE are at increased risk for premature diabetes and cardiovascular disease (1, 2) and AE has long been presumed to directly promote fatty liver disease in females. Initially, we hypothesized that DHT would induce a fatty liver phenotype, and we conducted extensive experiments to rigorously test this hypothesis. Using a hyperandrogenic, body weight matched female mouse model with intact ovaries, we show that increasing DHT doses (4–10 mm) do not induce MASLD under RD. Unexpectedly, we observed that DHT attenuated WD induced MASLD by reducing hepatic TG yet concurrently exacerbates hyperlipidemia. This protection occurs independently of hepatic AR and insulin signaling and is mediated via activation of GH-Stat5 pathway that suppresses Cd36 expression and FA uptake.

DHT with WD resulted in elevated circulating insulin levels, increased HOMA-IR, and impaired glucose tolerance. Despite this, hepatic insulin signaling did not differ between groups, with PTT and PEPCK (Pck1) and G6Pase proteins unchanged. Further, de novo lipogenesis related gene expressions were also unchanged (qPCR and RNA-seq). The protective effect on steatosis persisted even at 6 months of DHT treatment with WD (liver TG: Con-Veh- 64.07 ± 3.41 mg/g vs. Con-DHT- 53.39 ± 2.94 mg/g, *P* ≤ 0.05, unpublished). Furthermore, mice with conditional hepatic AR KO exhibited a similarly protected lipid profile under DHT treatment, indicating that DHT protects against MASLD through a hepatic AR independent pathway.

Multiomics analyses indicate that DHT masculinizes the liver by increasing male pattern genes via sex-specific GH signaling (40, 41), as exemplified by elevated *Serpina1e* (42) and *Mup* (30) transcripts in the liver and increased serum proteins. Basal GH levels were reduced, whereas GH responsiveness was enhanced in the liver, consistent with stronger STAT5 activation. Our data support a role for GH-STAT5 signaling in hepatic lipid regulation (43, 44) and further reinforce evidence that pituitary–hepatic crosstalk contributes to MASLD risk (45). Given that DHT alters GH pulsatility and hepatic GH receptor sensitivity (33, 35, 36), androgen driven shifts in GH dynamics may account for the coordinated transcriptome changes we observed.

We investigated downstream effectors mediating lipid handling in AE females. GH pulsatility regulates Cd36 transcription through nuclear pSTAT5 (44), and females typically show higher Cd36 expression than males (39). STAT5 can repress *Cd36* by directly binding its promoter (43) and by modulating sex biased transcriptional networks involving HNF6, BCL6, and CUX2 (46, 47), as well as pathways linked to PPARG and RXR signaling (44). DHT markedly reduced hepatic CD36 expression and TG levels, despite increased FFA levels in circulation under WD. This reduction in liver TG was consistent with a decrease in hepatic oleic acid (18:1), the most abundant fatty acid measured, and a key substrate required for TG synthesis (48, 49, 50). In HepARKO mice, DHT still reduced Cd36 expression and TG levels in liver, indicating that reduced MASLD phenotype does not depend on hepatocyte AR signaling. In contrast, continuous GH infusion, which feminizes the liver and abolishes GH pulsatility (25, 51), led to increased CD36 expression and hepatic TG accumulation. This pattern aligns with prior models in which impaired GH signaling promotes steatosis, including long-term GH overexpression (52) and GHR knockout models (53). As in our DHT treatment, pulsatile GH from daily bolus injections activates STAT5 and reduces hepatic lipid content (54, 55), further supporting the importance of GH signaling in maintaining normal hepatic lipid homeostasis. Short-term, 2-weeks AAV-mediated GH expression does not replicate these effects, likely due to the limited duration of AAV expression (56, 57). Continuous GH also failed to stimulate lipolysis, as indicated by the decrease in serum FFA in DHT + GH group, suggesting that the increased liver TG is due to increased lipid uptake. While the absence of a GH-only group is a limitation, our data suggest that such a group would likely respond similarly to the DHT + GH group since GH overrides DHT effects on liver lipid metabolism. Additionally, we did not perform detailed GTT in these continuous GH infused mice, as HOMA-IR values were unchanged, however, more comprehensive metabolic phenotyping would strengthen future studies. Overall, these findings indicate that pulsatile GH driven STAT5–CD36 signaling is a key mechanism regulating hepatic TG levels.

We observed increased circulating TG's accompanied by an increase in VLDL-TG secretion. However, key VLDL regulators such as MTP (58), and TM6SF2 (59) were not altered, suggesting alternate pathways may contribute to this phenotype. Carboxyesterases (Ces) are located in the ER and influence both lipid storage and mobilization (60); however, the mechanisms underlying the increased VLDL secretion observed in our study remain to be determined. The literature reflects a complex role for Ces3: liver specific or whole body Ces3 deletion increases hepatic TG (61, 62), whereas Ces3 deletion decreases hepatic TG and overexpression increases hepatic TG (63). In our study, however, *Ces3b* is elevated in WD-DHT mice despite reduced hepatic TG. This apparent discrepancy likely reflects broader DHT driven GH–STAT5 reprogramming of hepatic lipid pathways. In particular, coordinated changes across multiple Ces isoforms, alterations in *Slco1a1* and *Cyp7b1*, along with decreased CD36 may contribute to the observed shifts in hepatic TG, cholesterol, and bile acid metabolism (64, 65), warranting further study.

**Fig. 8:**
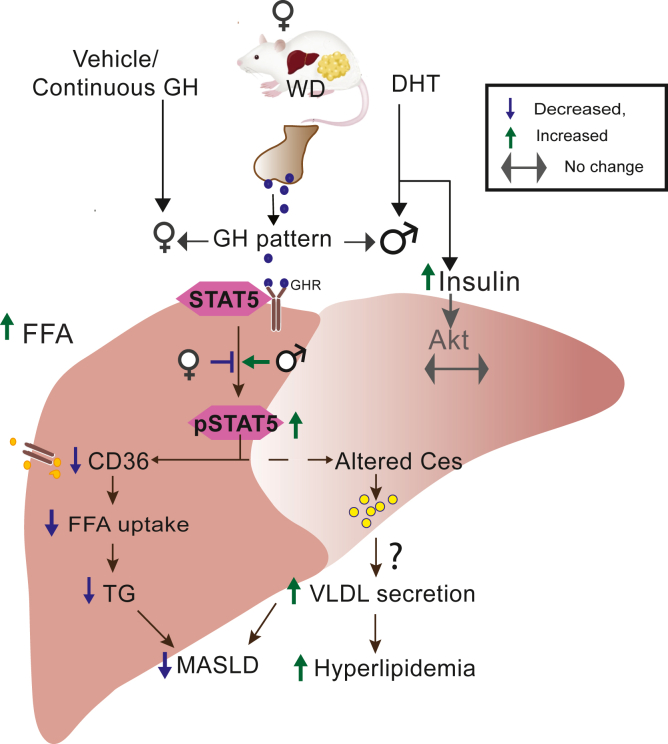
Androgen induced male pattern GH-STAT5 signaling protects the liver from lipid accumulation while promoting hyperlipidemia in female mice Female mice treated with DHT on WD exhibited increased serum insulin and FFA levels. In the liver, DHT imposed a male pattern GH signaling state marked by increased pSTAT5, which suppressed CD36 expression and reduced hepatocellular FFA uptake, thereby limiting TG accumulation. DHT altered Ces enzymes in the liver, likely augmenting VLDL-TG secretion and contributing to systemic hyperlipidemia. These findings highlight that DHT elicits organ specific metabolic effects, with impaired glucose homeostasis and enhanced lipolysis occurring alongside reduced hepatic lipid accumulation but increased TG secretion. WD, western diet; DHT, dihydrotestosterone; GH, growth hormone; FFA, free fatty acid.

Our comprehensive analysis of DHT effects on hepatic lipid metabolism under regular and high calorie diet, incorporating different doses and durations of DHT exposure, yielded results that differ from a previous report (66). That study reported DHT (4 mm) increased hepatic lipid accumulation under RD. However, the histological evidence (H&E staining) from the same study still showed minimal differences of lipid deposition between control and DHT treated mice (66). Hepatic steatosis is more common in obese women with PCOS, likely reflecting altered ectopic fat distribution driven by developmental factors such as intrauterine growth restriction (17, 18). Although males are generally more susceptible to liver disease and estrogens protect females (67, 68), our findings show that in adult onset AE females, androgen–GH interactions in the presence of intact ovaries recalibrate hepatic lipid handling. Collectively, these data indicate that AE induces hyperlipidemia while protecting the liver from steatosis (Fig. 8). These results highlight that adult onset AE differs fundamentally from early onset AE in hepatic lipid regulation.

## Data Availability

All data generated or analyzed during this study are included in this published article and its supplementary materials. The RNA-seq datasets generated during the current study have been deposited in the Gene Expression Omnibus (GEO) repository under accession number (G**SE326073)**

## Supplemental Data

This article contains supplemental data (69, 70, 71, 72, 73, 74, 75, 76, 77, 78).

## Conflict of Interest

The authors declare that they have no conflicts of interest with the contents of this article.
