# Supplementary material for: Androgen excess uncouples circulating and hepatic lipid homeostasis in females on a high-calorie diet
Source: J Lipid Res. 2026 Mar 30;67(5):101031. doi: 10.1016/j.jlr.2026.101031 (PMC13138190; doi:10.1016/j.jlr.2026.101031)
Supplement: Supplemental Tables S1–S7 and Figures S1–S6 [file mmc1.pdf]

Supplementary methodology

Metabolic Tolerance Tests

Whole-body glucose utilization, insulin sensitivity, and hepatic gluconeogenesis were evaluated using glucose tolerance tests (GTT), insulin tolerance tests (ITT), and pyruvate tolerance tests (PTT), respectively. GTT, PTT, and ITT were performed at 3, 5, and 7 weeks of post-DHT treatment, respectively, following established protocols [1]. For the GTT, mice were fasted for 17 hours and injected intraperitoneally (*ip*) with dextrose (2 g/kg BW). For the ITT, mice were fasted for 6 hours and injected *ip* with insulin (0.5 IU/kg BW). For the PTT, mice were fasted for 13 hours and injected *ip* with sodium pyruvate (2 g/kg BW). Blood glucose levels were measured at designated time points post-injection to assess glucose clearance, insulin sensitivity, and hepatic gluconeogenic capacity respectively.

Comprehensive Laboratory Animal Monitoring System (CLAMS)

To assess whole-body energy metabolism under conditions of calorie excess and elevated androgens, mice underwent indirect calorimetry using a Comprehensive Laboratory Animal Monitoring System (CLAMS; Columbus Instruments, Columbus, OH). Five to six weeks after initiation of DHT treatment and WD, mice were single housed in CLAMS cages and acclimatized for 12 hours. Metabolic parameters, including respiratory exchange ratio (RER), energy expenditure (heat production calculated as calorific value × VO2), ambulatory activity, and food intake, were continuously monitored over a 48-hour period under a 12-hour light/dark cycle. Mice were provided with an in-house crushed version of their assigned diet throughout the monitoring period [2].

Triglyceride and Cholesterol Determination

Fasting serum TG were measured using the Triglyceride Determination Kit (Sigma-Aldrich, Cat# TR0100, USA), following the manufacturers' protocols. For liver tissue TG measurements, accurately weighed liver samples were homogenized in 10 volumes of 5% NP-40 substitute (Research Products International, Cat# N59000-100, USA) according to previously published protocol [3, 4]. Homogenates were centrifuged at 4°C at 10,000 rpm for 10 minutes, the supernatant (including the upper buffy coat) was collected, and TG concentrations were measured at 540 nm wavelength. Serum and liver cholesterol were assessed using the Cholesterol Assay Kit (Abcam, Cat# ab65390, USA), according to the manufacturer's protocol.

VLDL TG Secretion

Hepatic TG production was assessed in 4 hour fasted mice injected i.p. with Poloxamer-407 (Sigma-Aldrich, Cat# 16758; 1,000 mg/kg) to inhibit lipoprotein lipase [5]. Blood was collected at 0, 1, and 2 h post injection, and serum TG measured using colorimetric assay (Sigma-Aldrich, Cat# TR0100) at 540 nm.

Fatty Acid Uptake

Liver pieces (~6 mm^3) were incubated with 1 μM BODIPY FL C16 (Thermo Fisher Scientific, Cat# D3821) in PBS with 0.25% BSA at 37 °C for 1.5 h in the dark, adapted from previous publications [6, 7]. Tissues were homogenized in RIPA buffer, and fluorescence (Ex/Em: 485/515 nm) was measured and normalized to protein. Fold changes were calculated relative to Con-Veh.

Histology

Mouse liver tissues (n=8-12) were fixed in 10% formalin, dehydrated in isopropanol and xylene, embedded in paraffin, and sectioned at 5 µm thickness using a microtome (Leica Biosystem, Germany) as previously described [8]. Paraffin-embedded liver sections were deparaffinized, rehydrated, and processed for various types of staining described below. 10 sections were used from each mouse for each staining methodology.

For Hematoxylin & Eosin (H&E) staining, sections were stained with hematoxylin, rinsed, counterstained with eosin, dehydrated, and cleared. For Sirius Red staining, sections were stained with picro-sirius red (prepared from Direct Red 80 (Sigma-Aldrich, Cat# 365548, USA) in picric acid solution)) for 1 hour and rinsed with acidified water. For F4-80 immunohistochemistry, sections underwent antigen retrieval using citrate buffer (Vector Labs, Cat. H3300, USA), were blocked with rabbit serum, and incubated overnight at 4°C with F4/80 antibody (1:2000, Proteintech Cat# 29414-1-AP, RRID:AB_2918300). The next day, sections were incubated with a biotinylated secondary antibody (Vector Laboratories Cat# PK-4001, RRID:AB_2336810) and developed using DAB (Vector Laboratories Cat# SK-4100, RRID:AB_2336382) followed by hematoxylin counterstaining.

For Oil Red O staining, liver tissues (n=8-12) were fixed in 4% paraformaldehyde, cryoprotected in 10% and 30% sucrose solutions consecutively, embedded in OCT compound, and sectioned at 7 µm using a cryostat (Epredia, CryoStar NX70). Sections were washed in distilled water, dehydrated with 60% isopropyl alcohol, stained with 60% Oil Red O solution (Sigma-Aldrich, Cat# O1391, USA), and mounted in an aqueous mounting medium before imaging under a Nikon microscope

After staining, all sections were cover slipped and imaged using a Nikon light microscope. For all staining methods, two representative images per section were captured from regions near the portal vein and central vein, and one image was taken from other regions. Image acquisition was performed blinded to the treatment group. Quantification of F4/80 staining was conducted using Fiji/ImageJ software, employing color deconvolution and DAB quantification as previously described [2].

RNA Sequencing and Bioinformatics Analysis

RNA sequencing was done by a commercial vendor (Azenta Life Sciences, New Jersey). Briefly, total RNA was extracted from mouse liver tissues (n= 4 each group) and RNA integrity assessed with the Agilent TapeStation 4200 (Agilent Technologies, Palo Alto, CA, USA). ERCC RNA Spike-In Mix (ThermoFisher Scientific, Cat. #4456740) was added to normalized RNA prior to library preparation. RNA libraries were prepared using the NEBNext Ultra II RNA Library Prep Kit for Illumina (NEB, Ipswich, MA, USA). Libraries were validated on the TapeStation, quantified, and sequenced on an Illumina NovaSeq (2 × 150 bp PE). The read depth was 20M reads per sample. Raw BCL files were converted to FASTQ and demultiplexed using bcl2fastq v2.20, allowing one mismatch. Adapter trimming and quality filtering were performed with Trimmomatic v0.36. Clean reads were aligned to the ENSEMBL reference genome using STAR v2.5.2b. Gene counts were obtained using feature Counts (Subread v1.5.2), counting only uniquely mapped reads in exon regions. Differential gene expression analysis was performed using DESeq2. Genes with adjusted p-values ≤ 0.05 and absolute log2 fold change (FC) ≥ 1 and ≤ -1 were used in constructing the volcano plot. For the purposes of biological pathway analysis, all genes with adj p value ≤ 0.05 were used. The networks, functional analyses were generated through the use of QIAGEN IPA (QIAGEN Inc., https://digitalinsights.qiagen.com/IPA), (October 2023 version) [9]. Gene ontology and pathway analysis were performed using Webgestalt (https://www.webgestalt.org/).

Serum proteomics

Serum proteins were extracted by diluting 5 μL of serum in 95 μL lysis buffer (4% SDS, 100 mM Tris pH 8) containing protease and phosphatase inhibitors, followed by BCA assay quantification and dilution to 0.5 mg/ml. Proteins were reduced with 5 mM DTT, alkylated with 50 mM iodoacetamide, and processed using filter-aided sample preparation (FASP) on 30 kDa MWCO filters with washes in 8 M urea and 50 mM ammonium bicarbonate. Digestion was performed overnight at 37C with trypsin (1:50 enzyme-to-protein ratio), quenched with 1% formic acid, and peptides dried. Samples were analyzed on an Orbitrap Eclipse mass spectrometer coupled to a nano LC system, using a 110-minute C18 gradient, operating in data-dependent acquisition mode with HCD fragmentation. Mass spectrometers were operated in positive ion full scan mode with data-dependent acquisition (DDA). The data were analyzed using Proteome Discoverer Version 3.1. Processing workflow using SequestHT as a search algorithm with a precursor mass tolerance of 5 ppm. Cysteine carbamidomethylation (+57.021 Da) was included as a static modification with methionine oxidation (+15.995 Da) included as a dynamic modification. Spectra were searched against the Mus musculus proteome (containing 25103 sequences). Protein quantification was done via summed abundance of unique + razor peptides.

Extraction and LC-MS method for the analysis of non-esterified fatty acids (NEFA/FFAs)

NEFA/FFAs were extracted from liver tissue following previously described methods [10]. Approximately 30 mg of liver tissue was weighed into an Eppendorf tube. To each sample, 250 μL of ice-cold extraction solvent (80% methanol/water) and 10 μL of internal standard ([13C16]-palmitic acid, 50 ng/μL) were added. The mixture was homogenized for 1 min using a Geno/Grinder and centrifuged at 20,000 × g for 10 min at 4 °C. A 50 μL aliquot of the resulting supernatant was transferred to an LC vial, and 3 μL was used for injection. NEFA analysis was performed using a Vanquish UHPLC system coupled to an Orbitrap Exploris 480 mass spectrometer (Thermo Fisher Scientific). Chromatographic separation was achieved at 25 °C using a hydrophilic interaction chromatography (HILIC) method with an Xbridge amide column (100 × 2.1 mm i.d., 3.5 μm; Waters). Mobile phase A: water with 5 mM ammonium acetate (pH 6.8), and mobile phase B: 100% acetonitrile. Linear gradient is: 0 min, 85% B; 1.5 min, 85% B; 5.5 min, 35% B; 6.9 min, 35% B;10.5 min, 35% B; 10.6 min, 10% B; 12.5 min, 10% B; 13.5 min, 85% B; 17.9 min, 85% B; 18 min, 85% B; 20 min, 85% B. The flow rate is: 0-5.5 min, 0.110 mL/min; 6.9-10.5, 0.130 mL/min; 10.6-17.9 min, 0.250 mL/min;18.0-20.0 min,0.110 ml/min. The parameters of Orbitrap Exploris 480 were as listed: vaporizer temperature, 150 °C; ion transfer tube temperature, 300 °C; sheath gas, 35; auxiliary gas, 7; sweep gas, 1; spray voltage,3.5 kV for positive mode and 3.0 kV for negative mode; RF-lens (%), 30; resolution was set at 60,000 (at m/z 200); scan range, 70-900 (m/z); positive/negative switching mode.

Data processing was performed using Sieve software (Thermo Fisher Scientific). NEFA species were identified based on theoretical MS1 m/z values and retention times of standard compounds. Integrated peak areas were determined from MS1 scans. The concentration of each fatty acid was calculated relative to the internal standard ([13C16] palmitic acid), and final fatty acid compositions were determined based on these concentration values.

References

1. Andrisse, S., et al., *Low-Dose Dihydrotestosterone Drives Metabolic Dysfunction via Cytosolic and Nuclear Hepatic Androgen Receptor Mechanisms.* Endocrinology, 2017. **158**(3): p. 531-544.
2. Ubba, V., et al., *Neuronal AR Regulates Glucose Homeostasis and Energy Expenditure in Lean Female Mice With Androgen Excess.* Endocrinology, 2023. **164**(11).
3. Stec, D.E., et al., *Loss of hepatic PPARα promotes inflammation and serum hyperlipidemia in diet-induced obesity.* Am J Physiol Regul Integr Comp Physiol, 2019. **317**(5): p. R733-r745.
4. Tong, L., et al., *PPARδ attenuates hepatic steatosis through autophagy-mediated fatty acid oxidation.* Cell Death & Disease, 2019. **10**(3): p. 197.
5. Millar, J.S., et al., *Determining hepatic triglyceride production in mice: comparison of poloxamer 407 with Triton WR-1339.* J Lipid Res, 2005. **46**(9): p. 2023-8.
6. Khalifeh-Soltani, A., et al., *Mfge8 promotes obesity by mediating the uptake of dietary fats and serum fatty acids.* Nat Med, 2014. **20**(2): p. 175-83.
7. Lee, J.H., et al., *A novel role for the dioxin receptor in fatty acid metabolism and hepatic steatosis.* Gastroenterology, 2010. **139**(2): p. 653-63.
8. Ubba, V., et al., *Reproductive Profile of Neuronal Androgen Receptor Knockout Female Mice with Low Dose of DHT.* Endocrinology, 2023.
9. Krämer, A., et al., *Causal analysis approaches in Ingenuity Pathway Analysis.* Bioinformatics, 2014. **30**(4): p. 523-30.
10. Scheidemantle, G., et al., *Data-dependent and -independent acquisition lipidomics analysis reveals the tissue-dependent effect of metformin on lipid metabolism.* Metabolomics, 2024. **20**(3): p. 53.

Suppl. Fig 1

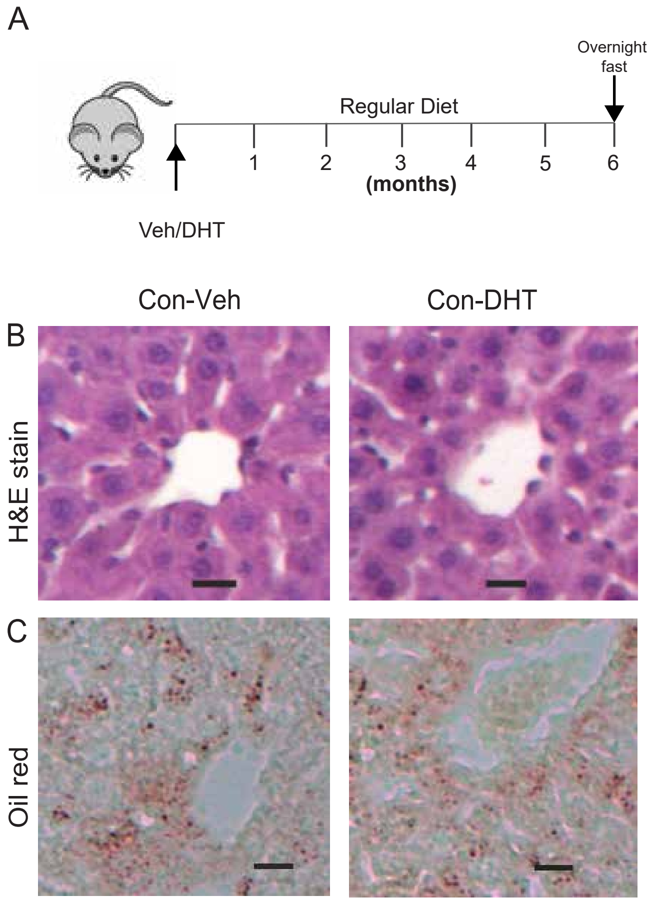

Suppl. Figure 1. DHT did not alter liver lipids in mice fed a regular diet

(A) Schematic Design: Ar floxed female mice on regular diet were treated with DHT (~3 fold vs Veh) or vehicle (Veh) for 6 months. (B-C) Representative H&E stain, Oil red stain images. Scale bar=20 μM.

Suppl. Fig 2

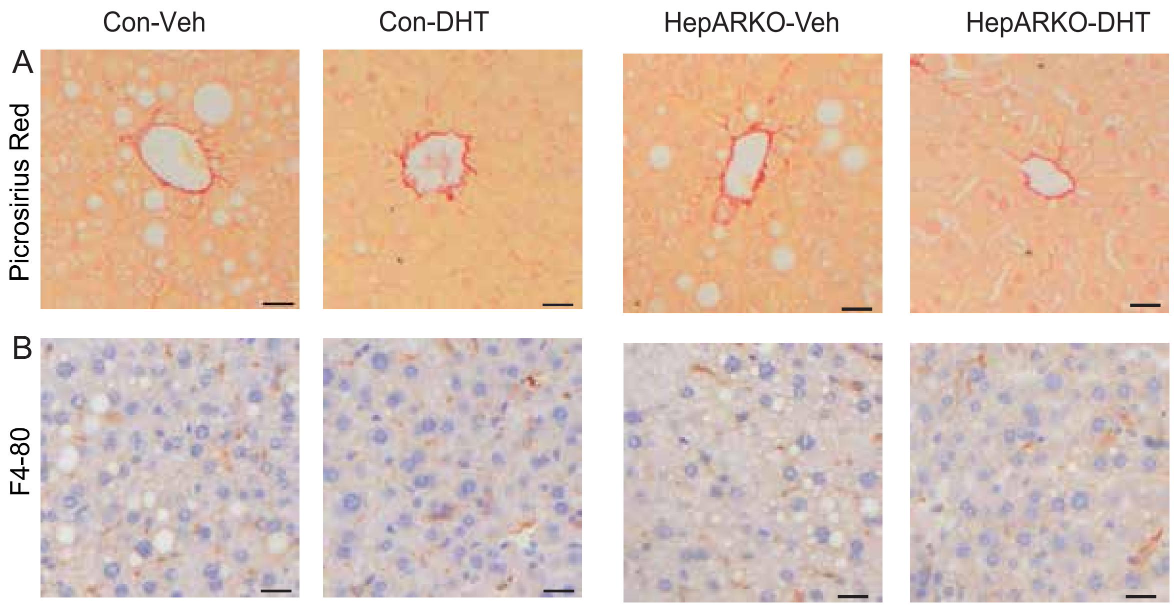

Suppl. Fig 2. DHT did not induce fibrosis or change inflammation in WD fed mice

(A-B) Representative Picrosirius red (20x), F4-80 (20x) staining in Con-Veh, Con-DHT mice, HepARKO-Veh, HepARKO-DHT. Scale bar: 20 μM

Suppl. Fig 3

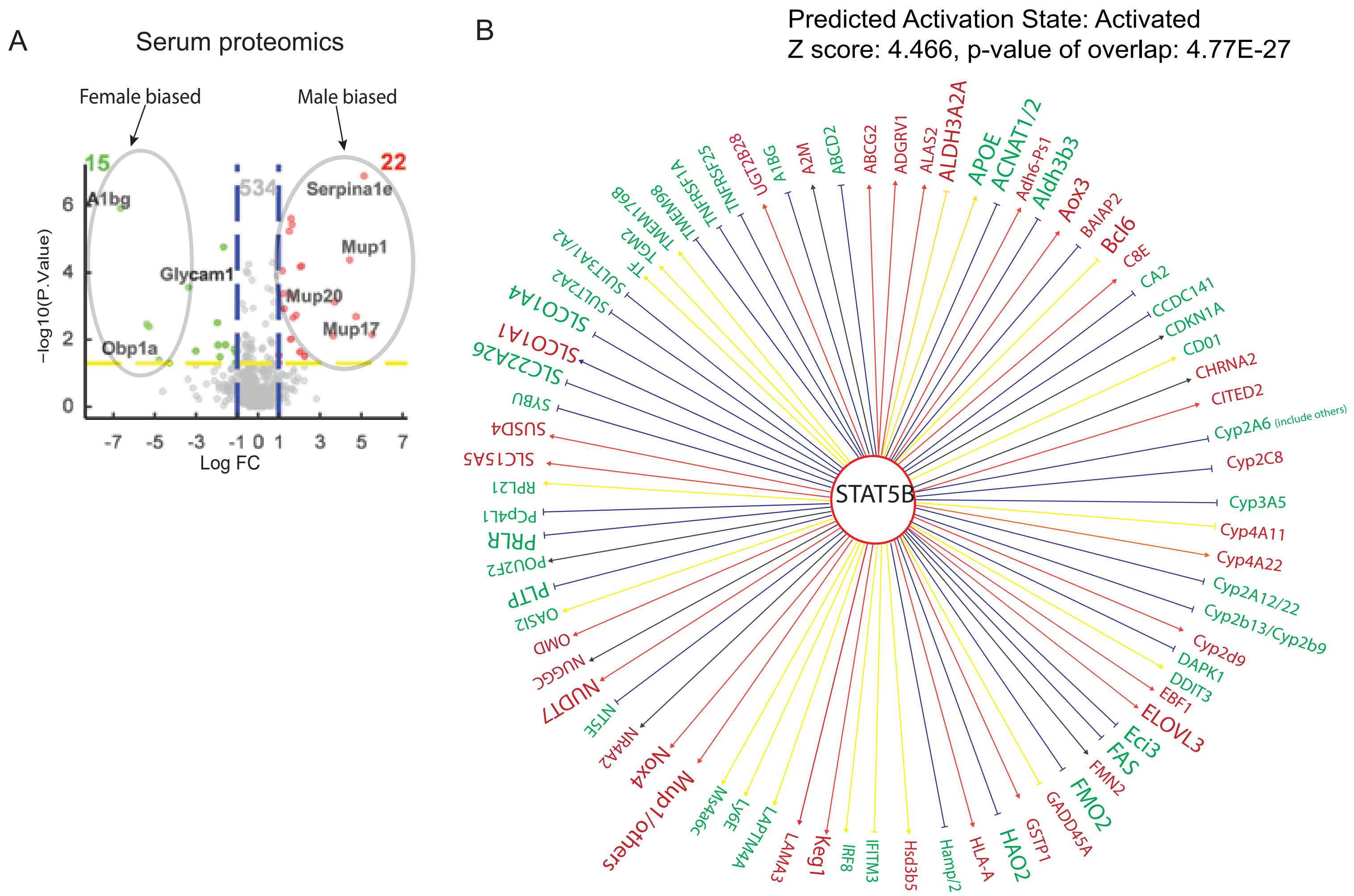

Suppl. Fig 3. Serum proteome and liver genes altered by Stat5 showed a male biased transcription pattern following DHT in female mice

(A) Volcano plot of differentially regulated proteins by DHT in serum proteome (N=4-6). Proteins are annotated by their respective gene names. (B) STAT5b was identified by IPA as top upstream activator from hepatic genes DEG (adj $p<0.05$) (N=4 per group). A pictorial representation of the genes mediated by STAT5b. Genes (nodes): green- down regulated, red- up regulated. Genes involved in metabolism are shown in bigger font. Arrows: orange- predicted activation, blue- predicted inhibition, yellow- inconsistent findings, grey line- not predicted.

Suppl. Fig 4

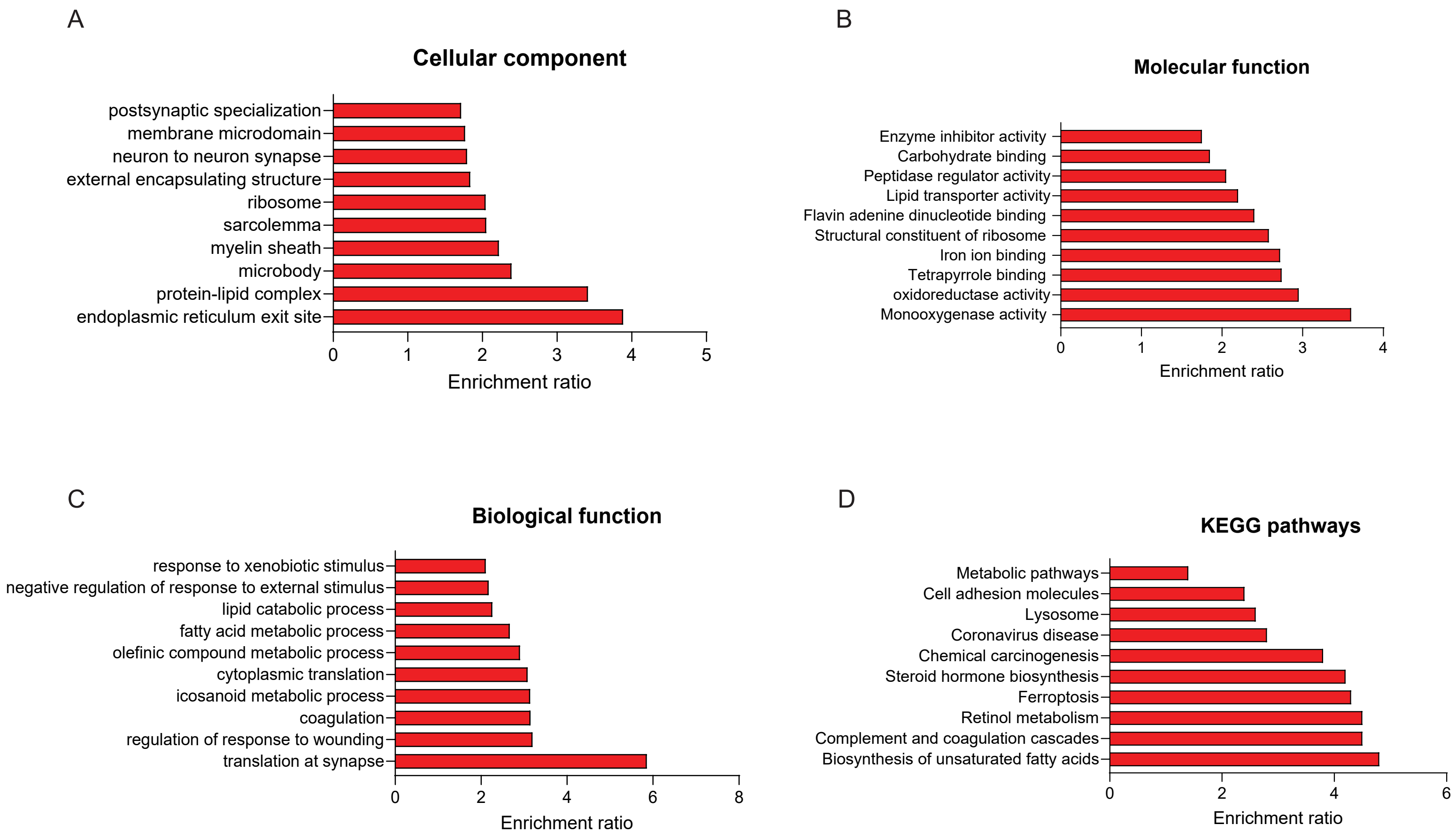

Suppl. Fig 4. Metabolic pathways are enriched in livers of DHT treated WD fed mice

(A) Cellular component, (B) Molecular function, (C) Biological function, (D) KEGG pathways of differentially regulated genes (both up and down regulated) following DHT treatment in livers of WD mice. Pathways were visualized by Webgestalt (https://www.webgestalt.org/).

Suppl. Fig 5

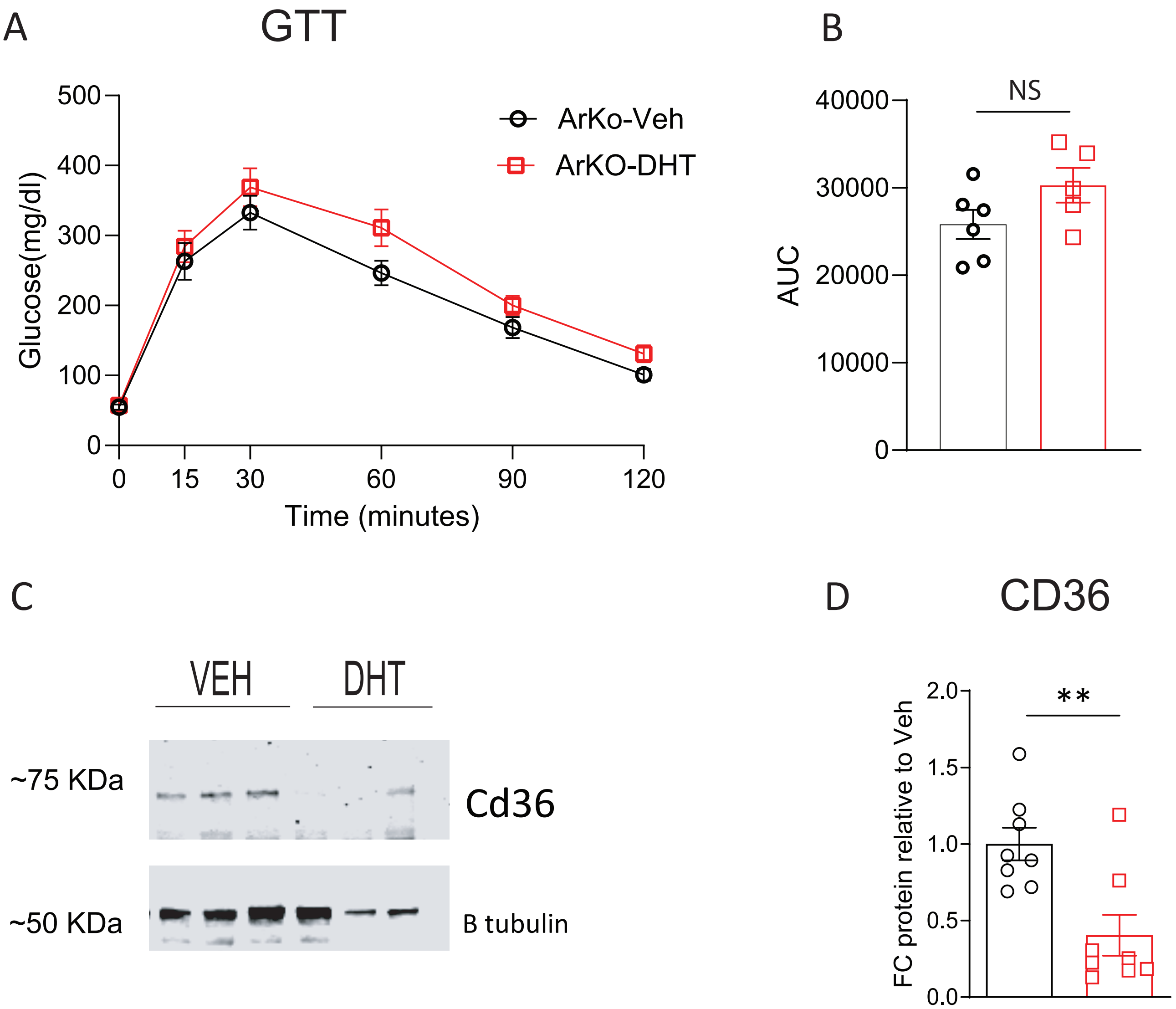

Suppl. Fig 5. DHT reduced CD36, a fatty acid transporter in livers of Hep ARKO mice.

(A) Glucose tolerance test (GTT), (B) Area under curve (AUC) for GTT. (C-D) Representative western images, densitometric analysis of CD36 in HepARKO-Veh abd HepARKO-DHT mice. (N=5-8). Statistical analysis was performed by Student t-test. Values are mean ± SEM. $p \le 0.05$; ** $p < 0.01$, NS- non significant. FC= Fold change

Suppl. Fig 6

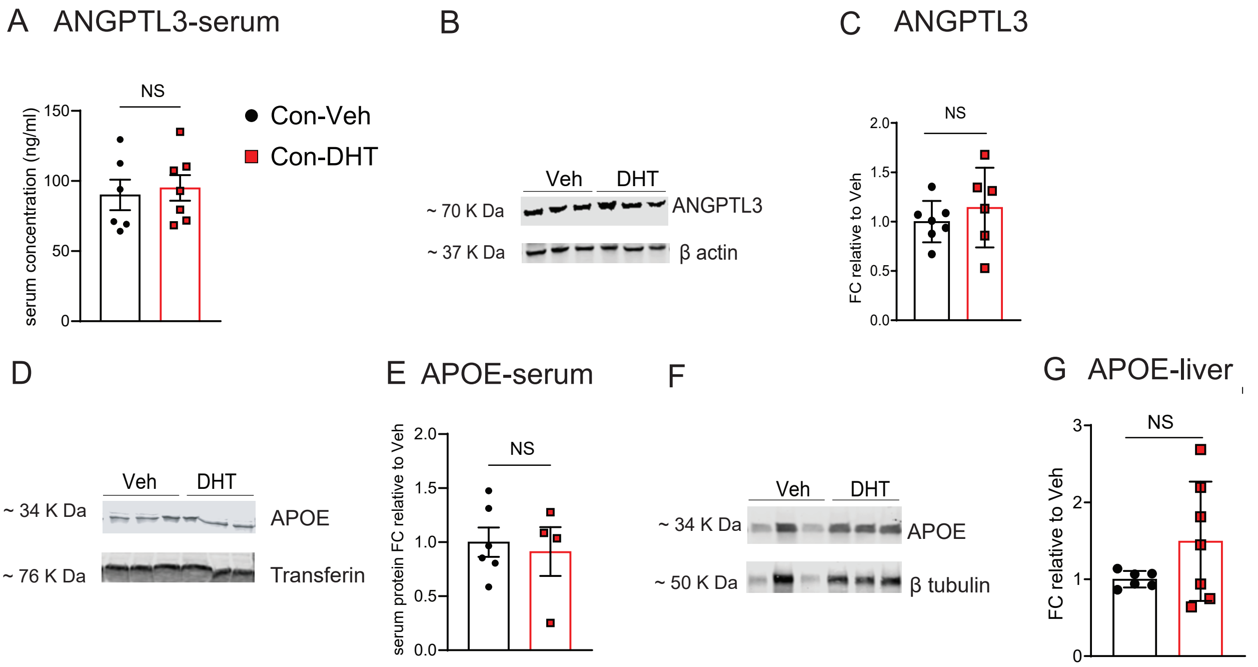

Suppl. Fig 6. Proteins involved in lipid secretion remain unchanged in DHT-treated WD-fed mice

(A)Serum Angptl3 was measured by ELISA. (B-C) Representative immunoblot of Angptl3 and its densitometric analysis in livers of mice fed WD. (D-E) Representative immunoblot, densitometric analysis of serum APOE.
(F-G) Representative immunoblot, densitometric analysis of APOE in livers of mice fed WD. (N=4-7)
Statistical analysis was performed using student t-test. Values are mean ± SEM. p≤0.05; NS- non significant.
FC= Fold Change

Suppl. Table 1

Analyte	Kit	Vendor	Catalog #	RRID
Insulin	Ultra-Sensitive Mouse Insulin ELISA Kit	Crystal Chem	90080	AB_2783626
Leptin	Leptin Mouse ELISA Kit	Thermo Fisher Scientific	KMC2281	
FGF21	Mouse/Rat FGF-21 Quantikine ELISA Kit	R&D Systems	MF2100	AB_2783730
DHT	Human Dihydrotestosterone (DHT) ELISA Kit	Alpha Diagnostic International	1940	AB_3678590
ALT	ALT/GPT Liquid (Kinetic) Assay	Teco Diagnostics	NC9851323	—
Prolactin	Milliplex Mouse Pituitary Magnetic Bead Panel	Millipore	RPTMAG-86K	AB_2716840
GH	Milliplex Mouse Pituitary Magnetic Bead Panel	Millipore	RPTMAG-86K	AB_2716840
Angptl3	Mouse ANGPTL3 ELISA Kit	Thermofisher	EMANGPTL3	AB_3716557

Suppl. Table 1. List of kits used in serum hormone and ALT assays

Suppl. Table 2

ACSL1 FP	CAGAACATGTGGGTGTCCAG
ACSL1 RP	GTTACCAACATGGGCTGCTT
FAS FP	GTCTGTGTCTTTTTGGACGGC
FAS RP	CAGCTTTCTCGGACTCCCAC
FATP2 (SLC27A2) FP	ATTCGTGCCAAGTCTCTGCT
FATP2 (SLC27A2) RP	GTAAAAGACGGACACGGCAT
FATp5 FATP5 (SLC27A5) FP	GCCTATGCCACACCTCATTT
FATp5 FATP5 (SLC27A5) RP	ACCCGGACAACTTTGTGAAG
CD36 FP	ATGGGCTGTGATCGGAACTG
CD36 RP	AGCCAGGACTGCACCAATAAC
LIPG FP	ATGCGAAACACGGTTTTCCTG
LIPG RP	TGGGTTTATGATGCTCATCTCG
CPT1a FP	CCATCCTGTCCTGACAAGGTTTAG
CPT1a RP	CCTCACTTCTGTTACAGCTAGCAC
APOE FP	CTGACAGGATGCCTAGCCG
APOE RP	CGCAGGTAATCCCAGAAGC
Apoa4 FP	CAGTGAGGAGCCCAGGATGTT
Apoa4 RP	TCTACAGCCTCCTTGGCATT
Ces3b FP	AAAATACCTTCGAGCCTTCCC
Ces3b RP	TTTCCTGTGCGTGCAAACT
SLCO1A1 FP	CAGGGGCATGCAGGATGTAT
SLCO1A1 RP	GTCTGGAGAGTGGATGTCGC
Igf1 FP	GCTGCTGAAGCCATTCATTT
Igf1 RP	TTGCTCTTAAGGAGGCCAAA
RPL19 FP	ATGAGTATGCTCAGGCTACAGA
RPL19 RP	GCATTGGCGATTTCATTGGTC

Suppl. Table 2. List of primers used in q-PCR.

Suppl. Table 3

Target	Host / Clone	Vendor	Catalog #	RRID
PCK1	Rabbit polyclonal	Abcam	Ab70358	AB_1925305
G-6-Pase	Rabbit polyclonal	Novus	NBP1-80533	AB_11044224
CD36	Rabbit monoclonal	Cell Signaling Technology	28109	AB_3675401
p-STAT5 (Y694)	Rabbit monoclonal	Cell Signaling Technology	4322	AB_10544692
Akt (pan)	Rabbit monoclonal	Cell Signaling Technology	4691	AB_915783
p-Akt (S473)	Rabbit monoclonal	Cell Signaling Technology	9271	AB_329825
β-Actin	Mouse monoclonal	Thermo Fisher Scientific	MA5-15739	AB_10979409
Lamin A/C	Mouse monoclonal	Cell Signaling Technology	4777	AB_10545756
HDAC1	Mouse monoclonal	Abcam	Ab68436	AB_1860585
Angptl3	Rabbit polyclonal	Thermo Fisher Scientific	MA5-35681	AB_2849581
ApoE	Rabbit polyclonal	Cell Signaling Technology	49285	AB_3714592
β-tubulin	Mouse monoclonal	Thermo Fisher Scientific	MA5-16308	AB_2537819
Transferrin	Rabbit monoclonal	Proteintech	17435-1-AP	AB_2035023

Suppl. Table 3. List of antibodies used in western blots

Suppl. Table 4: Adult female mice receiving DHT for 6 months did not have changes in liver and serum lipids.

Regular Diet 6 months		
	Veh	DHT
Bw (g)	28.14 ± 0.83 [a]	28.03 ± 0.58 [a]
Corrected liver weight (g/g)	0.043 ± 0.000 [a]	0.041 ± 0.001 [a]
liver TG (mg/g)	28.81±3.21 [a]	25.14±2.05 [a]
Liver Cholesterol (mg/g)	3.39±0.44 [a]	3.80±0.64 [a]
Serum TG (mg/dL)	60.34±7.10 [a]	63.76±3.91 [a]
Serum Cholesterol (mg/dL)	129.4±8.98 [a]	115.7± 8.40 [a]
ALT (U/L)	53.81± 13.35 [a]	40.79± 3.51[a]

Data were analyzed using Student's *t*-tests (N=6-15). Values are mean ± SEM

Suppl. Table 5: Adult female mice fed with WD and receiving DHT for 8.5 weeks show increased brown adipose tissue weight (corrected to body weight).

	Con-Veh	Con-DHT	P value
g/g	Mean ±SEM	Mean ±SEM	
GF	0.008±0.002	0.010±0.001	NS
IF	0.007±0.001	0.008±0.001	NS
BAT	0.0018±0.0001	0.0023±0.0001	**

Data were analyzed using Student's *t*-tests (N=10-11). Values are mean ± SEM ($p \leq 0.05$). **- $p<0.01$, NS-Non Significant.

Suppl. Table 6: Top 20 Upregulated and Downregulated Hepatic Genes Identified by RNA-seq

Top 20 downregulated genes				
	Gene.name	log2FoldChange	P value	adjusted p-value
1	Fmo3	-8.05	2.08E-37	1.49E-33
2	Cyp2b13	-6.66	1.74E-21	8.32E-19
3	Sult3a1	-6.34	3.82E-19	1.41E-16
4	Sult2a2	-6.23	9.12E-06	0.00029
5	A1bg	-5.85	1.32E-08	9.33E-07
6	Cyp3a44	-5.77	2.6E-25	2.07E-22
7	Slc22a26	-5.75	2.1E-18	7.17E-16
8	Gm29920	-5.73	2.63E-09	2.25E-07
9	Cyp2a4	-5.60	1.62E-24	1.16E-21
10	Cyp3a16	-5.45	1.78E-05	0.000511
11	Cyp3a41a	-4.74	4.12E-05	0.001035
12	Gm42375	-4.62	1.34E-08	9.37E-07
13	Aldh3b3	-4.34	4.82E-20	2.04E-17
14	Rsph4a	-4.06	1.43E-12	2.23E-10
15	Cyp2c69	-3.99	5.24E-07	2.47E-05
16	Hao2	-3.99	7.72E-26	7.92E-23
17	Slc22a27	-3.31	8.72E-33	3.13E-29
18	Cyp2b9	-3.12	0.000341	0.005887
19	Lcn2	-3.06	0.00477	0.045325
20	Prtn3	-3.05	9.35E-05	0.002032
Top 20 Upregulated genes				
	Gene.name	log2FoldChange	P value	adjusted p-value
1	Mup7	9.05	2.65E-33	1.27E-29
2	Cyp4a12a	8.08	1.63E-25	1.47E-22
3	Cyp4a12b	7.45	3.20E-27	4.17E-24
4	Mup14	6.82	2.89E-28	5.19E-25
5	Mup1	6.60	1.09E-20	4.74E-18
6	Hsd3b5	6.31	1.61E-10	1.67E-08
7	Mup11	6.07	1.48E-26	1.64E-23
8	Pitx3	5.88	1.87E-07	9.85E-06
9	Obp2a	5.69	2.49E-10	2.47E-08
10	Mup20	5.25	3.91E-103	5.61E-99
11	Mup16	5.12	4.26E-14	9.27E-12
12	Csn3	5.04	2.93E-06	0.000111
13	Mup-ps19	4.85	5.61E-30	1.15E-26
14	Mup12	4.81	4.33E-16	1.20E-13
15	C330002G04Rik	4.52	1.07E-30	2.56E-27
16	Mup17	4.49	1.77E-10	1.81E-08
17	Serpina1e	4.42	5.68E-05	0.001358
18	Cyp2d9	4.40	1.53E-10	1.61E-08
19	Mup15	4.21	3.45E-09	2.87E-07
20	Gm3839	3.96	3.16E-05	0.000833

RNA-seq was conducted on liver tissue from Veh and DHT mice fed a WD (N = 4 per group). Differentially expressed genes were identified using DESeq2, and the top 20 upregulated and top 20 downregulated genes are shown, ranked by $\log_2$ fold change. Log2FC and adj P values were generated using Wald test.

Suppl. Table 7: Significant Differential Regulation of Carboxylesterase Family Members

	log2FC	P value	adjusted p-value
Ces1c	-0.71715	8.87E-18	2.77E-15
Ces3b	1.308827	2.67E-10	2.63E-08
Ces3a	0.844658	1.56E-07	8.28E-06
Ces1b	-0.94278	5.78E-07	2.68E-05
Ces2c	1.656466	5.96E-05	0.001416
Ces1g	-0.75902	0.001341	0.017825
Ces4a	2.775824	0.001764	0.022155

RNA-seq analysis conducted on liver tissues from Veh and DHT mice (N=4 per group). Data revealed significant differential expression of multiple carboxylesterase (Ces) genes. Log2FC and adj P values were generated using Wald test.
